# Macronutrient balance determines the human gut microbiome eubiosis: insights from *in vitro* gastrointestinal digestion and fermentation of eight pulse species

**DOI:** 10.3389/fmicb.2024.1512217

**Published:** 2025-01-30

**Authors:** Da Bin Lee, In Seon Hwang

**Affiliations:** Food and Nutrition Division, Department of Agri-food Resources, National Institute of Agricultural Sciences, Wanju, Republic of Korea

**Keywords:** gut fermentation, *in vitro* digestion, human microbiome, pulse, diversity

## Abstract

The interactions between macronutrients, the human gut microbiome, and their metabolites (short-chain fatty acids) were comprehensively investigated via an *in vitro* digestion and fermentation model subjected to eight pulse species. 16S rRNA sequencing and taxonomic analysis of pulse digesta fermented for up to 24 h revealed an increase in the relative abundance of gut health-detrimental genera represented by *Escherichia-Shigella* in kidney bean, soybean, cowpea, chickpea, and black bean samples. In contrast, the relative abundance of health-positive genera, including *Bacteroides*, *Eubacterium*, and *Akkermansia*, was elevated in red bean, mung bean, and Heunguseul. At the same time, the proportion of the pathogenic *Escherichia-Shigella* decreased. Concurrently, these three species exhibited an increase in microbial diversity as evidenced by the calculation of *α*-diversity (Shannon index) and *β*-diversity (Bray-Curtis distance). Despite the lower nutrient contents in the three pulses, represented by carbohydrates, amino acids, and fatty acids, network analysis revealed that the nutrient contents in the pulse digesta possess complex positive or negative correlations with a variety of bacteria, as well as their metabolites. These correlations were more pronounced in red bean, mung bean, and Heunguseul than in the other pulses. It was postulated that the overall potential to nourish gut environments in these species was due to the balance of their nutritional components. The linear regression analysis demonstrated that there was a negative association between carbohydrate and amino acid contents and the increase in Shannon indices. Furthermore, the ratio of carbohydrates to fatty acids and amino acids to fatty acids displayed negative correlations with the diversity increase. The ratio of carbohydrates to amino acids showed a weak positive correlation. It is noteworthy that a diet comprising foods with a balanced nutritional profile supports the growth of beneficial gut microbes, thereby promoting microbial eubiosis. Consistent work on different ingredients is essential for precise insight into the interplay between food and the human microbiome in complex dietary patterns.

## 1 Introduction

In response to the growing efforts of various industries to achieve carbon neutrality amidst climate change, the food industry has witnessed a notable surge in demand for plant-based diets as alternatives to conventional animal-derived foods (Sandua, 2024). In comparison to animal-based foods, those derived from plants are rich in phytochemicals, micronutrients, and dietary fibers. Additionally, they contain minimal amounts of saturated fats and trans fats, which have been linked to adverse effects on cardiovascular health (Gong et al., 2020). However, plant-based foods have certain limitations. For instance, they often have restricted amount of micronutrients that are predominantly found in meat or seafood, as well as a lower quantity and quality of protein (Tso and Forde, 2021).

To address these shortcomings, pulses have been employed extensively as a pivotal ingredient in plant-based diets. In addition to their high protein and dietary fiber content, pulses contain beneficial unsaturated fatty acids, such as omega-3, which qualifies them as a nutritionally valuable food source (Karolkowski et al., 2021; Didinger and Thompson, 2022). Globally, pulse products are widely consumed, with notable examples including tofu, natto, tempeh, and soy sauce. Given the recent interest in the gut microbiome as a key factor for maintaining human health, there is a growing recognition of the potential of fermented foods made from pulses to nurture the growth of beneficial bacteria, including *Streptococcus* sp. and *Faecalibacterium* sp. (Valentino et al., 2024). This is attributed to the presence of prebiotic components, such as polysaccharides, and probiotic species that are abundant in these foods.

To elucidate the precise relationship between food and the microbiome, it is essential to identify the impacts of each ingredient, before the combinations of foods, on the growth or reduction of gut bacteria. One of the limiting factors of exploiting microbiome-informed precision nutrition strategies is the difficulty of identifying the effect of a single food in our complex dietary intake (Valles-Colomer et al., 2023). As complex individual diets in clinical studies hinder the comprehension of the effects of specific dietary components on the gut microbiota, dietary metadata is needed for the advancement of microbiome-targeted therapies (Ross et al., 2024). However, there is a paucity of knowledge regarding how food ingredients, in addition to a single nutrient or dietary pattern, influence the gut microbiota. A number of recent studies have consistently reported that the intake of dietary fibers and polysaccharides from food consumption (Deehan et al., 2020; Coker et al., 2021) or certain diet types, such as the Mediterranean diet (Meslier et al., 2020; Rinott et al., 2022), can improve the diversity of the human microbiome and support the proliferation of beneficial bacteria, thereby exerting positive health effects.

This approach presents a challenge in current practices due to the inherent limitations of the self-reporting dietary assessment technique, which is dependent on an individual’s memory (Johnson et al., 2020). To overcome these challenges and assess the specific impact of individual ingredients on the human microbiome, an *in vitro* digestion and colonic fermentation method was developed by multiple researchers (Xavier and Mariutti, 2021). This system simulates the digestive process of food through the human mouth, upper gastrointestinal tract, and colonic fermentation process under controlled conditions, thereby providing standardized results in a time- and cost-effective manner compared to human trials. Consequently, the system allows for the rapid and accurate evaluation of the effects of a variety of daily consumed ingredients on the gut microbiota. As the INFOGEST protocol has been validated for *in vitro* digestion studies of foods, it has been utilized in current studies examining a range of food items, such as leafy vegetables and pulses (Lee et al., 2019; Kim et al., 2021; Bae et al., 2021). Meanwhile, the final destination of consumed food, gut fermentation, has not been sufficiently investigated in the majority of research, which has been limited to the simulation of the digestion process and the measurement of the bioaccessibility of food ingredients. The combined model of *in vitro* digestion and gut fermentation system allows for the prediction of the ultimate health effects of food consumption.

This study aimed to investigate the impact of commonly consumed pulses in Korea on the human gut microbiota and metabolite profiles. Pulses constitute a principal component of the Korean diet, with a diverse range of pulses being consumed in Korea. In this study, the following pulses were selected as analytic samples: soybean, kidney bean, cowpea, black bean, red bean, mung bean, Heunguseul, and chickpea. The last one is a non-domestic species whose consumption has recently increased, though it is not cultivated in Korea. The digested products of pulses prepared using an *in vitro* digestion model were cultured in an *in vitro* gut fermentation model simulating the human colon environment to observe the effects on gut microbiota. Modifications were made to the INFOGEST protocol in order to accurately simulate the digestion of nutrients. These included the addition of the BBMV (brush border membrane vesicles) enzyme, which was extracted from the porcine small intestine and subjected in the final stage of digestion. Furthermore, given that pulses are a food source that typically contains macronutrients (carbohydrates, proteins, and fats), the study also analyzed the interrelationships between these components in pulses and changes in the gut microbiota, as well as their metabolites. This was done to ascertain the nutritional factors that contribute to the prebiotic effects of pulse samples.

## 2 Materials and methods

### 2.1 Preparing pulses in edible form

Eight types of pulses commonly consumed in Korea were selected for analysis as follows: soybeans (*Glycine max*, SB), kidney beans (*Phaseolus vulgarius*, KB), cowpeas (*Vigna unguiculata*, CB), chickpeas (*Cicer arietinum*, CP), black beans (*Rhynchosia nulubilis*, BB), mung beans (*Vigna radiate*, MB), Heunguseul (*Vigna anaularis L. Heunguseul*, HS), and red beans (*Vigna anaularis L.*, RB). The samples were prepared in a consumable form by applying the most common cooking methods suggested by the National Standard Food Composition Table (provided by the Rural Development Administration, 10th version). Consequently, each sample was subjected to either boiling or stir-frying, with the specific methods outlined as follows: The SBs (Sangju, Gyeongsangbuk-do, Korea) were boiled for 40 min in water at three times their weight after a 4-hour soaking. CBs (Gurye, Jeollanam-do, Korea) were boiled for 50 min in water at 10 times their weight after soaking for 8 h. The KBs (Geochang, Gyeongsangnam-do, Korea), HSs (Wanju, Jeollabuk-do, Korea), MBs (Muan, Jeollanam-do, Korea), and RBs (Muan, Jeollanam-do, Korea) were boiled for 1 h in water at 10 times of their weight without prior soaking. The CP samples (USA) were boiled for 15 min in water at 10 times their weight, following a 6-hour soaking. The roasted BBs (Sangju, Gyeongsangbuk-do, Korea) were obtained from a commercial market.

### 2.2 Simulated digestion using *in vitro* gastrointestinal model

A gastrointestinal model was constructed and simulated to examine the alteration of nutrient contents throughout the digestive process, which encompasses the oral, gastric, intestinal, and BBMV phases (Minekus et al., 2014). In the oral phase, cooked pulses were mixed with a 1:1 solution of simulated salivary fluid (KCl (Potassium chloride P9333, Sigma Aldrich Co, Seoul, Korea) 15.1 mmol/L, KH_2_PO_4_ (Potassium phosphate monobasic P0662, Sigma Aldrich Co.) 3.7 mmol/L, NaHCO_3_ (Sodium bicarbonate S5761, Sigma Aldrich Co.) 13.6 mmol/L, MgCl_2_(H_2_O)_6_ (Magnesium chloride hexahydrate 63,068, Sigma Aldrich Co.) 0.15 mmol/L, (NH_4_)_2_CO_3_ (Ammonium carbonate 207,861, Sigma Aldrich Co.) 0.06 mmol/L, pH 7.0) and enzyme (75 U/mL salivary *α*-amylase solution (α-Amylase from human saliva A0521, Sigma Aldrich Co.), 0.3 M CaCl_2_ (Calcium chloride C1016, Sigma Aldrich Co.), water). The solution was digested at 37°C for 2 min. The gastric phase entailed the mixture of the oral digested samples and a 1:1 solution of simulated gastric fluid (KCl 6.9 mmol/L, KH_2_PO_4_ 0.9 mmol/L, NaHCO_3_ 25 mmol/L, NaCl (Sodium chloride S9888, Sigma Aldrich Co.) 47.2 mmol/L, MgCl_2_(H_2_O)_6_ 0.1 mmol/L, (NH_4_)_2_CO_3_ 0.5 mmol/L, pH 3.0) and enzyme (2000 U/mL porcine pepsin stock solution (Pepsin from porcine gastric mucosa P7000, Sigma Aldrich Co.), 0.3 M CaCl_2_, 1 M HCl (Hydrochloric acid 4095–3705, Daejung, Seoul, Korea), water). The solution was incubated at 37°C for 120 min. Similarly, the intestinal phase was conducted through the mixing of gastric digested samples with a 1:1 solution of simulated intestinal fluid (KCl 6.8 mmol/L, KH_2_PO_4_ 0.8 mmol/L, NaHCO_3_ 85 mmol/L, NaCl 38.4 mmol/L, MgCl_2_(H_2_O)_6_ 0.33 mmol/L, (NH_4_)_2_CO_3_ 0.6 mmol/L, pH 7.0) and enzyme (200 U/mL pancreatin from porcine pancreas [Pancreatin from porcine pancreas P7545, Sigma Aldrich Co.]), 160 mM bile salts (Bile extract porcine B8631, Sigma Aldrich Co.), 0.3 M CaCl_2_, water and reacting at 37°C for 120 min. As the final digestive phase, 77.3 U/mL of BBMV was added for the digesta to react at 37°C for 180 min (Oku et al., 2011). Subsequently, the mixture was heated at 100°C for 10 min to inhibit further digestion.

### 2.3 Dialysis process

The samples that underwent *in vitro* digestion were collected and placed in a 1 kDa molecular weight cut-off cellulose membrane (Spectra/Por^®^ 6 Standard RC Pre-Wetted Dialysis Tubing). They were then soaked in 10 mM NaCl at 37°C for 24 h to remove low-molecular-weight components (Shiowatana et al., 2006; Bouayed et al., 2011; Hettiarachchi et al., 2021). Once the dialysis process was complete, the digested material was transferred to the *in vitro* gut fermentation model.

### 2.4 Determination of the nutrient content in digested pulses

The cooked pulses were subjected to oral, gastric, small intestinal digestion, and dialysis phases, and the major nutrient contents of each fully digested pulse sample were analyzed. A total of six types of saccharides, including oligosaccharides, disaccharides, and monosaccharides, were determined. In addition, 22 essential and non-essential amino acids and 6 fatty acids were quantified. These nutrients serve as energy sources for the gut microbiota in the simulated human gut, and the analysis data were used to examine the correlation between nutrient content and microbial growth.

#### 2.4.1 Analysis of free saccharides in digested samples

The levels of six free saccharides were determined for the pulse samples treated by the digestion and dialysis process. All samples were filtered (0.45 μm) and were analyzed using a Dionex Ultimate 3,000 (Thermo Dionex, Germany) and Sugar-pak (Waters, 300 mm × 6.5 mm) column. Standards for glucose (Junsei chem, 98%), galactose (Sigma, 99%), arabinose (Aldrich, 99%), xylose (Aldrich, 99%), fructose (Sigma, 99%), mannose (Sigma, 99%), sucrose (Sigma, 99.5%), maltose monohydrate (Junsei chem, 99%), lactose monohydrate (Junsei chem, 99%), raffinose (Sigma, 99%), and stachyose (Sigma, 99%) were used.

#### 2.4.2 Analysis of free amino acids in digested samples

Twenty-two amino acids were quantified for the digested samples. They were diluted with a buffer (0.1 M perchloric acid, 0.1% meth-phosphoric acid) for ultrasonic extraction for 1 h and were homogenized for 1 h at room temperature. Samples were filtered (0.2 μm) and were analyzed on a Dionex Ultimate 3,000 (Thermo Dionex, Germany) and Inno C18 column (Younjin biochrom, 4.6 mm × 150 mm, 5 μm). Standards for aspartic acid (Asp, Agilent 5061-3330), glutamic acid (Glu, Agilent 5061-3330), serine (Ser, Agilent 5061-3330), histidine (His, Agilent 5061-3330), glycine (Gly, Agilent 5061-3330), threonine (Thr, Agilent 5061-3330), alanine (Ala, Agilent 5061-3330), arginine (Arg, Agilent 5061-3330), GABA (Sigma), taurine (Tau, Sigma), tyrosine (Tyr, Agilent 5061-3330), valine (Val, Agilent 5061-3330), methionine (Met, Agilent 5061-3330), phenylalanine (Phe, Agilent 5061-3330), isoleucine (Ile, Agilent 5061-3330), leucine (Leu, Agilent 5061-3330), lysine (Lys, Agilent 5061-3330), proline (Pro, Agilent 5061-3330), cysteine (Cys, Agilent 5061-3330), glutamine (Gln, Agilent 5062-2478), asparagine (Asn, Agilent 5062-2478), and tryptophan (Trp, Agilent 5062-2478) were used.

#### 2.4.3 Analysis of free fatty acids in digested samples

The amounts of six fatty acids (palmitic acid, stearic acid, arachidic acid, oleic acid, linoleic acid, *α*-linolenic acid [Supelco 37 Component FAME Mix, Supelco, United States)] were determined. Freeze-dried samples were mixed with 340 μL of methylation mixture (MeOH: benzen: DMP: H_2_SO_4_ = 39:20:5:2) and 200 μL of heptane for extraction at 80°C for 2 h. The supernatants were analyzed at room temperature using an Agilent 7890A GC–MS (Agilent, USA) and a DB-23 column (Agilent, 60 mm × 0.25 mm × 0.25 mm). FID (280°C, H2 35, Air 350, He 35 mL/min) was used as the detector.

### 2.5 Quantification of human microbiome abundance and metabolite contents

#### 2.5.1 *In vitro* human gut fermentation

The *in vitro* batch fermentation process was performed according to the method established by Mandalari et al. (2008). The fecal slurry used to simulate the human intestinal environment was the mixture of fresh feces from 10 healthy adults who had not consumed prebiotics or probiotics for 6 months prior to sample collection (approved by the Institutional Review Board of the National Institute of Agricultural Sciences, BR-202307-01). The fecal slurry diluted with 0.1 M PBS at pH 7.0 was left in an anaerobic condition for a period of time, after which the supernatant was injected into the culture medium. The fermentation medium was prepared with peptone water (2 g/L), yeast extract (1 g/L), NaCl (0.1 g/L), K_2_HPO_4_ (0.04 g/L), KH_2_PO_4_ (0.04 g/L), MgSO_4_·7H_2_O (0.01 g/L), CaCl_2_·2H_2_O (0.01 g/L), NaHCO_3_ (2 g/L), bile salts (0.5 g/L), L-cysteine hydrochloride (0.5 g/L), hemin (50 mg/L), vitamin K_1_(10 μL/L), and Tween 80 (2 mL/L). After 15 mL of the fecal slurry was injected into 135 mL of the medium, pulse digesta was added to make 1% (w/v) of the total mixture, which was fermented under anaerobic conditions maintained by the oxygen-free nitrogen gas (15 mL/min). The fermentation was maintained at pH 6.7 at 37°C, and samples were retrieved every 0, 12, and 24 h. Non-substrate samples were produced as controls in which the pulse digesta was replaced with the distilled water. Additionally, fecal samples collected without the pretreatment process were analyzed for their microbiome abundance to verify that the *in vitro* model functioned appropriately and reflected the characteristics of the original fecal microbiota.

#### 2.5.2 16S amplicon and shotgun metagenome sequencing for taxonomy analysis

Microbial sequencing and taxonomy profiling were performed by Macrogen, Inc. (Seoul, Korea) and Life Mining Lab., GIST (Gwangju, Korea). DNA was extracted using a DNeasyPowerSoil Kit (Qiagen, Germany) according to the manufacturer’s instructions. The extracted DNA was quantified using Quant-IT PicoGreen (Invitrogen).

For 16S analysis, the sequencing libraries were prepared according to the Illumina 16S Metagenomic Sequencing Library protocols to amplify the V3 and V4 regions. The input gDNA 2 ng was PCR amplified with 5× reaction buffer, 1 mM of dNTP mix, 500 nM each of the universal F/R PCR primer, and Herculase II fusion DNA polymerase (Agilent Technologies, United States). The cycle condition for the first PCR was 3 min at 95°C for heat activation, and 25 cycles of 30 s at 95°C, 30 s at 55°C and 30 s at 72°C, followed by a 5-min final extension at 72°C. The universal primer pair with Illumina adapter overhang sequences used for the first amplifications were as follows: V3-F: 5′-TCGTCGGCAGCGTCAGATGTGTATAAGAGACAGCCTACGGGNGGCWGCAG-3′, V4-R: 5′- GTCTCGTGGGCTCGGAGATGTGTATAAGAGACAGGACTACHVGGGTATCTAATCC-3′. The first PCR product was purified with AMPure beads (Agencourt Bioscience, USA). Following purification, the 2 μL of the first PCR product was PCR amplified for final library construction containing the index using NexteraXT Indexed Primer. The cycle condition for the second PCR was the same as the first except for 10 cycles. The PCR product was purified with AMPure beads. The final purified product was then quantified using qPCR according to the quantification protocol guide (KAPA Library Quantification kits for IlluminaSequencing platforms) and qualified using the TapeStation D1000 ScreenTape (Agilent Technologies, Germany). The paired-end (2 × 300 bp) sequencing was performed by the Macrogen using the MiSeq™ platform (Illumina, USA). After sequencing, Cutadapt (v3.2) was utilized to remove adapter and primer sequences from the raw data, with forward and reverse reads trimmed to 250 bp and 200 bp. To generate Amplicon Sequence Variants (ASV) sequences, the reads were performed error-correction, merging, and denoising processes with DADA2 (v1.18.0). Sequences with expected errors of two or more were excluded. Erroneous reads were denoised based on an established error model. Following error correction, paired-end reads were merged by overlapping. Chimeric sequences were eliminated using the consensus method with the removeBimeraDenovo function in DADA2. ASVs with lengths shorter than 350 bp were filtered out using R (v4.0.3). The resulting ASVs were utilized for downstream analysis using QIIME (v1.9.0). Each ASV was aligned to the organism with the highest similarity in the corresponding Reference Database (NCBI_16S), using algorithms such as Bayesian classifier (DADA2_v1.18.0, confidence value: 50). Alpha and beta diversity metrices were calculated to elucidate the complexity and compositional changes of microbial genus both within and between individual samples. Alpha diversity was employed to quantify the richness and evenness of microbial communities within each sample, while beta diversity was utilized to assess the differences in microbial composition across samples.

For three pulse samples that exhibited a significant increase in genus-level diversity, additional species-level analysis was conducted using Shotgun method. The sequencing libraries were prepared according to the manufacturer’s instructions of TruSeq Nano DNA High Throughput Library Prep Kit (Illumina). Briefly, 100 ng of genomic DNA was sheared using adaptive focused acoustic technology (Covaris) and the fragmented DNA is end-repaired to create 5′-phosphorylated, blunt-ended dsDNA molecules. Following end-repair, DNA was size selected with bead-based method. These DNA fragments go through the addition of a single ‘A’ base, and ligation of the TruSeq DNA UD Indexing adapters. The products are then purified and enriched with PCR to create the final DNA library. The libraries were quantified using qPCR according to the qPCR Quantification Protocol Guide (KAPA Library Quantification kits for Illumina Sequencing platforms) and qualified using the Agilent Technologies 4200 TapeStation D1000 screentape (Agilent technologies). Then we sequenced using the NovaSeq (Illumina). DNA Illumina HiSeq/NovaSeq raw data was demultiplexed by index sequences, and paired-end FASTQ files were generated for each sample after sequencing. Adapter sequences and data with an average phred quality score less than 20 were removed using Trimmomatic (v0.39) of the Kneaddata (v0.10.0) pipeline (option: SLIDINGWINDOW:4:20). Then, bowtie2 (v2.4.5) was used to remove the human genome sequences using the hg37dec_v0.1 reference (*Homo sapiens* reference genome). The pre-processed data were analyzed using MetaPhlAn4 (v4.0.0) for ~1 million microorganisms composed of NCBI reference genomes and species-level genome bins (SGBs). The reads were mapped to the specific marker genes of the microbial species, and the species abundance was calculated based on the average number of reads mapped to the marker genes. At this time, marker genes that mapped to less than 33% were removed as the species were considered absent. The taxonomic composition derived from the species abundance was presented by Krona (Ondov et al., 2011), a tool for calculating the relative proportions (%) of species within each microbial genus. This analysis served as a reference for the subsequent discussions regarding the potential health implications associated with specific microbial genera.

#### 2.5.3 Analysis of short-chain fatty acids (SCFAs) as microbial metabolites

Acetic acid, butyric acid, and propionic acid in the pulse digesta after intestinal fermentation were determined using HPLC Ultimate3000 (Thermo Dionex, United States) and RI detector (ERC, RefractoMAX520, Japan) at UV 210 nm. 10 μL aliquots of 0, 12, and 24 h fermentation samples were injected onto an ICSep Coregel 87H3 column (7.8 mm × 300 mm, concise separations, United States). The mobile phase was 0.01 N H_2_SO_4_ at a flow rate of 0.5 mL/min and an oven temperature of 40°C. Volatile organic acid mixture (AccuStandard FAMQ-004, 10 mM) containing formic, acetic, propionic, isobutyric, butyric, isovaleric, valeric, isocaproic, caproic, and heptanoic acid was the standard material for the analysis.

### 2.6 Statistical approach

All experiments were conducted in triplicate, and data entry, descriptive statistics, and the construction of bar graphs of the relative abundance of genera for each sample were performed using Microsoft Excel 2016 (Microsoft Corporation, Redmond, Washington, United States). All statistical tests, analyses, and visualizations, except those practiced by Excel were performed using RStudio (2023.12.1. +402 for Windows). To analyze differences in relative microbial abundances during colonic fermentation of digested pulse, a Wilcoxon rank-sum test (at the 95% confidence level) was used. For comparisons of *α*-diversity and *β*-diversity, the Shannon index (SI) was calculated for 0, 12, and 24 h fermentation samples of each pulse sample, and Bray-Curtis distances were used for multidimensional scaling (MDS) visualization. Significant differences in the nutrient contents and the SIs between samples were tested by ANOVA and Tukey’s method (*p* < 0.05) and presented in a box plot. Principal component analysis (PCA) was performed to identify the distribution of nutrient content—carbohydrates, amino acids, and fatty acids—within the digested pulse samples, selecting principal components with an accumulated variance greater than 80% and generating a biplot expressing loadings and scores. Primary variables for the network analysis were chosen from the factor analysis conducted on the carbohydrates, amino acid, and fatty acid content, as well as on the difference in absolute microbial abundance between 0 h and 12 h samples. Variables with a loading of ≥0.5 were selected, and only edges with a correlation >0.5 were included in the positive correlation network, while edges with a correlation < −0.5 were included in the negative correlation network. The centrality of each variable in the network analysis was calculated and represented by the size of the nodes. Communities within the network were identified using the Infomap method and displayed in different colors.

## 3 Results

### 3.1 Free carbohydrate, amino acid, and fatty acid contents in digested pulses

**Figure 1:**
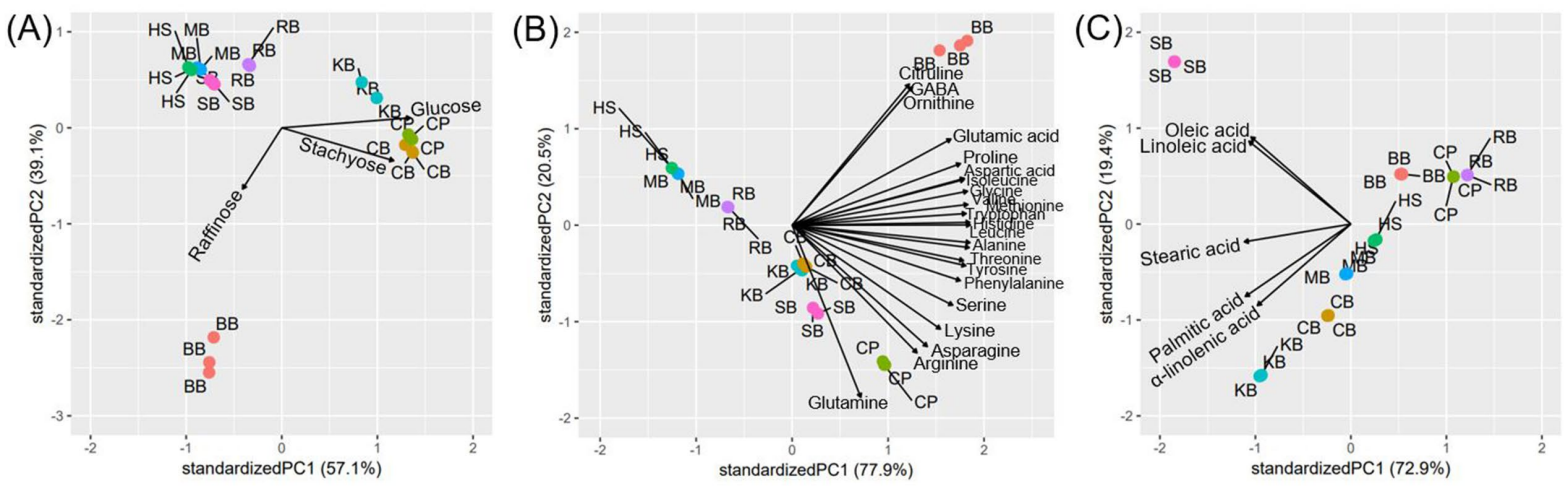
PCA biplot of the content of free saccharides **(A)**, amino acids **(B)**, and fatty acids **(C)** contents in the digested samples of eight pulses.

The degraded forms of carbohydrates in the pulses subjected to digestion were quantified, followed by the oral, gastric, small intestinal phases, and dialysis phases (Supplementary Table S1). During the digestive process, carbohydrates with high molecular weight were broken down into monosaccharides, and most of the low molecular weight compounds were eliminated by dialysis. However, some undigested oligosaccharides, such as stachyose, remained in the samples. The stachyose content in the digested samples of CB, CP, and BB was 63.84 ± 1.80 μg, 58.56 ± 0.99 μg, and 42.17 ± 0.45 μg, respectively, while 9.14 ± 0.57 μg of raffinose remained only in the digested BB. The monosaccharide glucose was detected in the digested samples of KB, CP, and CB at concentrations of 246.77 ± 1.93 μg, 228.95 ± 1.24 μg, and 206.96 ± 0.77 μg, respectively. Based on the PCA biplot (Figure 1A), samples other than KB, CP, CB, and BB contained relatively lower amounts of free saccharides.

Free amino acid content was also examined after proteins were degraded during the digestion process and subsequently filtered through dialysis phases. The consistent directionality of the loading vectors on the PCA biplot (Figure 1B) corresponding to each amino acid suggests a positive correlation in amino acid content across the samples. Notably, the loading vectors for glutamine, ornithine, and citrulline deviated slightly from the direction observed for other amino acids, indicating the possibility of differential levels of these amino acids in some pulses. Overall amino acid levels in eight pulses were high at glutamine, arginine, phenylalanine, and leucine (Supplementary Table S2). Specifically, glutamine showed particularly high levels, with contents of 92.05 ± 3.93 μg, 30.76 ± 0.13 μg, 23.80 ± 0.26 μg, 23.53 ± 0.36 μg, 20.54 ± 0.19 μg in the digested samples of BB, CP, CB, SB, and KB. Arginine also showed high concentrations of 27.60 ± 0.18 μg and 19.99 ± 0.23 μg in the digested CP and SB samples, while phenylalanine was present at levels of 21.76 ± 0.19 and 17.57 ± 0.73 in the CP and BB samples. Similarly, leucine was found in high concentrations of 27.62 ± 1.19 μg, and 23.39 ± 0.09 μg in BB and CP digesta, indicating that CP and BB digesta contained the highest overall levels of amino acids. As confirmed by the PCA plot (Figure 1B), relatively lower levels of amino acids were observed in samples from RB, KB, MB, and HS. Ornithine and citrulline were exclusively detected in the digested BB sample.

For free fatty acids, they were largely eliminated after dialysis, with only trace amounts remaining in the samples. According to the PCA plot (Figure 1C), the digested samples of BB, RB, and CP had the lowest fatty acid contents. In contrast, the digested SB sample contained the highest levels of oleic acid (10.02 ± 0.01 μg) and linoleic acid (25.27 ± 0.01 μg) (Supplementary Table S3). The digested KB sample also had a relatively high level of free fatty acids, with palmitic acid (3.67 ± 0.00 μg) and *α*-linolenic acid (5.51 ± 0.01 μg) present at higher levels compared to the other pulses.

### 3.2 Microbial taxonomic analysis of *in vitro* fermentation samples

**Figure 2:**
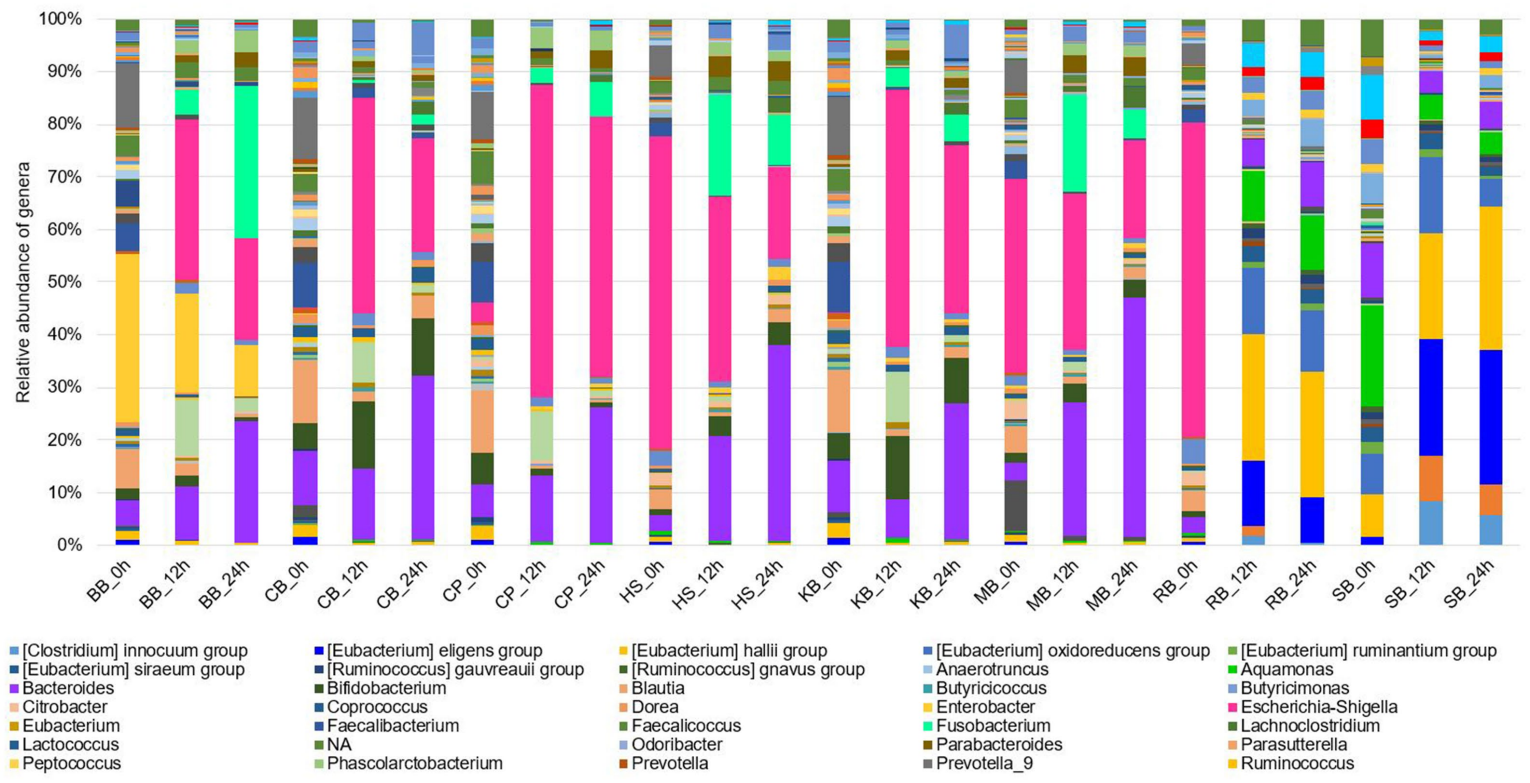
Composition of the gut microbiota of *in vitro* fermentation samples (0 h, 12 h, 24 h) of eight pulses at the genus level.

The eight different digested pulses were cultured using an *in vitro* gut fermentation model, and products were collected at 0, 12, and 24 h in each pulse digesta. After collection, the genus-level 16S (V3-V4) rRNA sequencing and taxonomic profiling were performed, and the relative abundance of microbes was visualized in a bar plot chart (Figure 2). In the initial (0 h) colonic fermentation of the BB digesta, *Enterobacter* represented approximately 40% of the total gut microbiota, while the *Prevotella 9* lineage accounted for around 15%. However, as the fermentation progressed, both *Enterobacter* and *Prevotella 9* decreased in their portion, while *Escherichia-Shigella*, *Fusobacterium*, and *Bacteroides* increased, resulting in a simplified microbial community.

Besides, in the CB digesta, the initial microbial composition displayed a distribution of the diverse genus, including *Prevotella 9*, *Faecalibacterium*, *Blautia*, and *Bacteroides*, each representing about 15% of the community. But, over fermentation time, *Escherichia-Shigella*, *Bifidobacterium*, and *Bacteroides* dominated the community. This trend of simplification of the microbial composition was particularly pronounced in the CP digesta, with a remarkable increase in *Escherichia-Shigella* and *Bacteroides*, which came to account for a large portion of the microbial community. Similar patterns were observed during the fermentation of the KB digesta, where the relative abundance of *Bifidobacterium* increased.

Although the SB digesta initially contained a diverse community, with *Alistipes*, *Akkermansia*, *Bacteroides*, *Aquamonas*, and various *Eubacterium* groups comprising a substantial proportion, the *Eubacterium* groups increasingly constituted the community as fermentation progressed. In particular, *Akkermansia* gradually decreased during fermentation. Overall, a common trend was observed in the fermentation of BB, CB, CP, and KB digesta, characterized by an increase in the relative abundance of *Escherichia-Shigella*, *Bacteroides*, and *Clostridium sensu stricto 1*, along with a decrease in *Blautia*.

In the fermentation of HS digesta, a marked decrease in the relative abundance of *Escherichia-Shigella*, which comprised about 60% of the primary microbial community, was observed over time. Concurrently, the proportions of *Bacteroides*, *Fusobacterium*, and *Bifidobacterium* increased. A similar pattern was found in the fermentation of MB digesta, with a decline in the relative abundance of *Bacillus* and an increase in *Bifidobacterium*. An inverse relationship between the relative abundances of *Escherichia-Shigella* and *Bacteroides* was identified in most pulse digesta.

During the fermentation of RB digesta, the relative abundance of *Escherichia-Shigella* decreased significantly, while the portions of *Bacteroides* and the *Eubacterium* groups increased. This trend was consistent with the results seen in the SB digesta where *Eubacterium oxidoreducens*, *Eubacterium hallii*, and *Eubacterium eligens* groups collectively accounted for approximately 45% of the microbial community at the 12 h and 24 h time points. Furthermore, unlike the other pulses, RB digesta showed an increase in the relative abundance of *Aquamonas*, *Alistipes*, and *Akkermansia*. While *Aquamonas* is prevalent in environmental materials such as water and has not been studied in the context of human health or food samples, the positive health effects of *Alistipes* and *Akkermansia* are described in the discussions of Table 1.

**Table 1:** The relative abundance of genera which exhibited a significant increase at 12 or 24 h of fermentation compared to the initial time point (0 h) for three pulse samples.

	RB	HS	MB
Genus^a^	%	%	%
Bacteroides	6.94 ± 1.62	28.57 ± 8.51	35.44 ± 10.02
Bifidobacterium	0.04 ± 0.01	4.07 ± 0.42	3.42 ± 0.05
Fusobacterium	0.06 ± 0.01	14.43 ± 4.76	11.84 ± 6.72
Parabacteroides	ND^b^	3.83 ± 0.26	3.63 ± 0.02
Parasutterella	ND^b^	0.10 ± 0.00	0.08 ± 0.02
Phascolarctobacterium	ND^b^	2.21 ± 0.29	2.04 ± 0.06
Sutterella	ND^b^	2.80 ± 0.18	2.57 ± 0.26
Alistipes	4.54 ± 0.30	0.60 ± 0.34^c^	0.66 ± 0.15
Butyricimonas	0.09 ± 0.00^c^	0.10 ± 0.02	0.08 ± 0.00^c^
Enterobacter	0.11 ± 0.01	1.74 ± 0.69	0.35 ± 0.29^c^
Coprococcus	–	0.77 ± 0.45^c^	0.76 ± 0.19^c^
Odoribacter	ND^b^	0.08 ± 0.02^c^	0.27 ± 0.11
Akkermansia	2.11 ± 0.30^c^	0.09 ± 0.01	0.30 ± 0.01
Lachnoclostridium	ND^b^	1.59 ± 1.30^c^	2.48 ± 1.32

a Listed all genus taxa in RB or HS or MB that had a significant increase in relative abundance after 12 or 24 h of fermentation compared to 0 h (Wilcoxon’s rank-sum test, *p* < 0.05).

b ND, Not Detected.

c There was no significant increase in the relative abundance at 12 or 24 h in comparison to 0 h.

**Figure 3:**
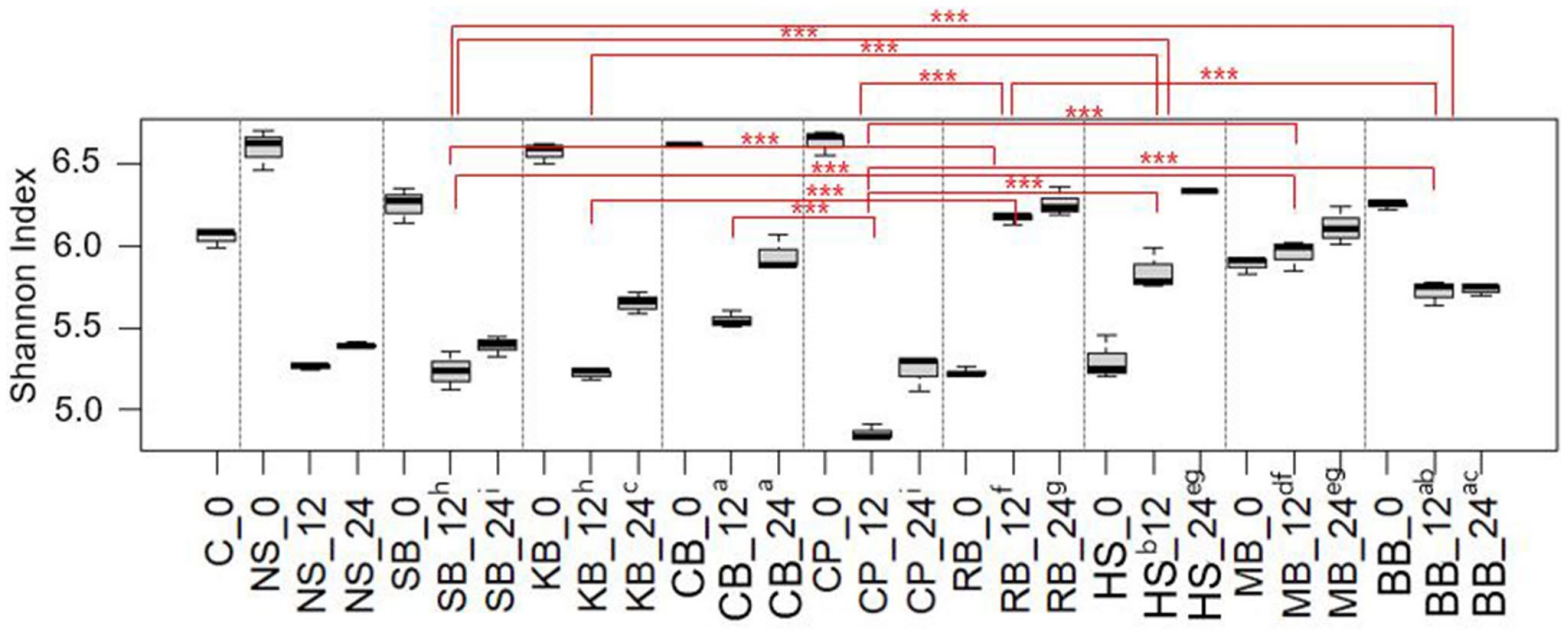
The change in Shannon indices of each pulse sample over fermentation hour (0 h, 12 h, 24 h). Samples with significant differences in Shannon indices at both 12 h and 24 h fermentation were connected by red lines and are indicated with their significance levels (^***^p < 0.001). Samples without significant differences were designated with the same alphabet. The C (control) and NS (non-substrate) samples were excluded from the significance test.

Furthermore, diversity analyses were conducted to provide a quantitative framework the patterns of microbial variability among the samples that were illustrated in the aforementioned bar plot. The ɑ-diversity (SI) of the pulse samples at each *in vitro* fermentation time was calculated to assess the genus complexity of the fecal microbiota within each pulse (Figure 3). In most samples, there was a decrease in diversity at 12 h of colonic fermentation compared to 0 h, with a slight increase observed at 24 h. However, in RB, HS, and MB, diversity consistently increased over time. The mean SIs at 0 h and 12 h for each sample were as follows: SB 6.25 and 5.24, KB 6.57 and 5.22, CB 6.60 and 5.54, CP 6.63 and 4.86, and BB 6.24 and 5.72, with a similar range of decrease. In contrast, the diversity increased from 5.23 to 6.17 in RB, from 5.30 to 5.84 in HS, and from 5.89 to 5.95 in MB, indicating an upward trend in these three pulses.

**Figure 4:**
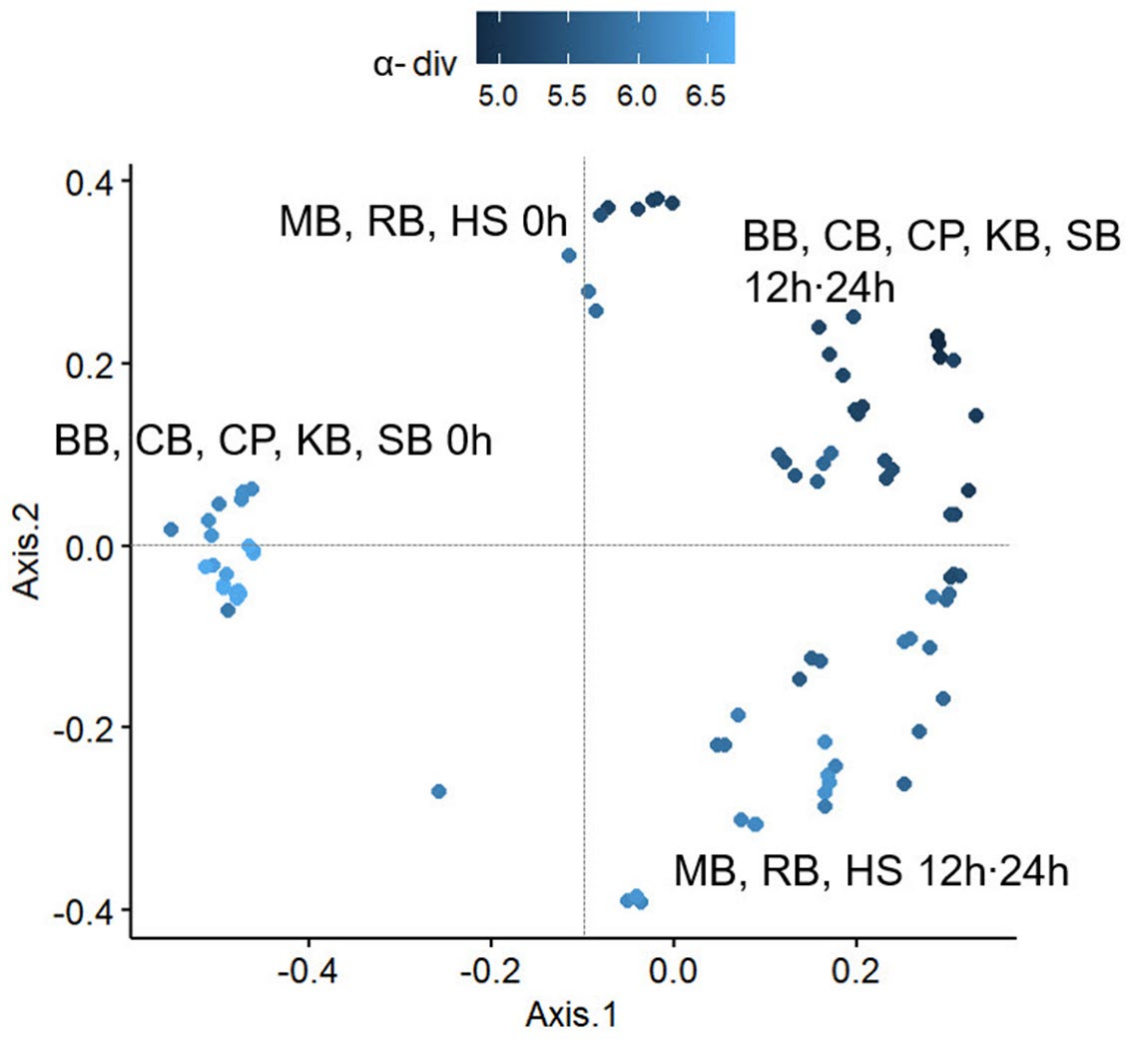
The plot based on a multidimensional scaling (MDS) analysis of the Bray-Curtis distances between pulse samples taken at each fermentation hour (0 h, 12 h, 24 h). BB, black bean; CB, cowpea; CP, chickpea; HS, Heunguseul; KB, kidney bean; MB, mung bean; RB, red bean; SB, soybean.

The *β*-diversity of the microbial communities was analyzed using Bray-Curtis distances between samples (Figure 4). Based on the calculated distances, a general distribution was observed by the MDS method, which exerted significant shifts in the composition of the gut microbiota at either 12 or 24 h of fermentation compared to 0 h in all pulse samples. Since KB, CB, CP, BB, and SB showed a decrease also in *α*-diversity as fermentation progressed, it was suggested that the digestive residues of these samples reduced the richness of the microbial distribution in the colon, simultaneously altering the composition of the microbiota. However, RB, HS, and MB digesta positively influenced the distribution of a variety of microorganisms during colonic digestion.

**Figure 5:**
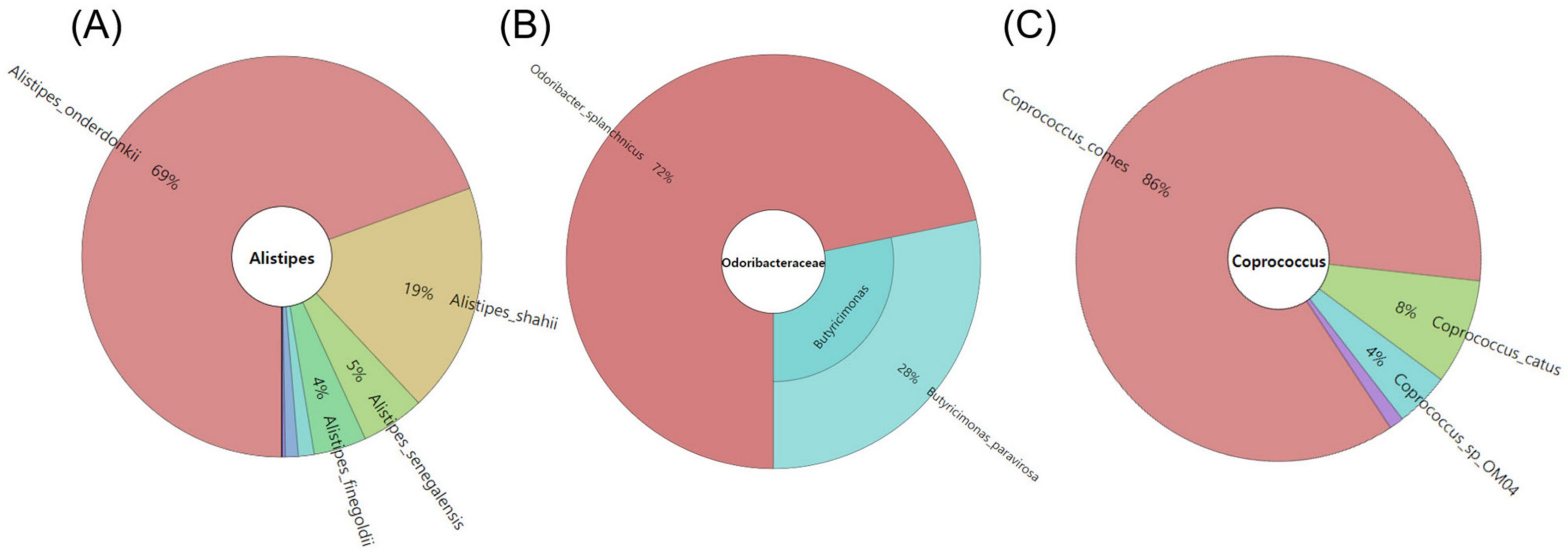
The taxonomic classification of species within the genus demonstrated a significant increase in relative abundance during the fermentation of HS **(A)**, MB **(B)**, and RB **(C)** digesta.

Changes in gut microbiome genera were observed in three pulses where health-positive bacteria increased during colonic digestion (Table 1). As the relative abundances of *Bacteroides*, *Bifidobacterium*, and *Fusobacterium* were found to be increased by colonic fermentation in all three samples, the relative growth of *Coprococcus* and *Lachnoclostridium* was uniquely depicted in RB, while *Odoribacter* and *Akkermansia* were specifically increased in MB. The species-level analysis performed by Shotgun whole genome sequencing for these genera (Figure 5) showed that these genera contained only *O. splanchnicus* and *A. muciniphila*, respectively. Meanwhile, the proportions of *Alistipes* and *Lachnoclostridium* increased in both RB and MB. *Alistipes* belongs to a relatively recently discovered sub-branch genus within the phylum Bacteroidetes. The Shotgun sequencing identified the following four species for *Alistipes*: *A. onderdonkii* (69%), *A. shahii* (19%), *A. senegalensis* (5%), and *A. finegoldii* (4%).

The relative abundances of *Sutterella* and *Akkermansia* also increased in MB and HS, while the relative growth of *Enterobacter* was promoted in RB and HS. In the case of *Enterobacter*, only *E. hormaechei* constituted a minute fraction, comprising 0.003% of the entire Enterobacteriaceae family.

### 3.3 Comprehensive correlations between nutrients, microbiome growth, and metabolite production

#### 3.3.1 Positive correlation networks

**Figure 6:**
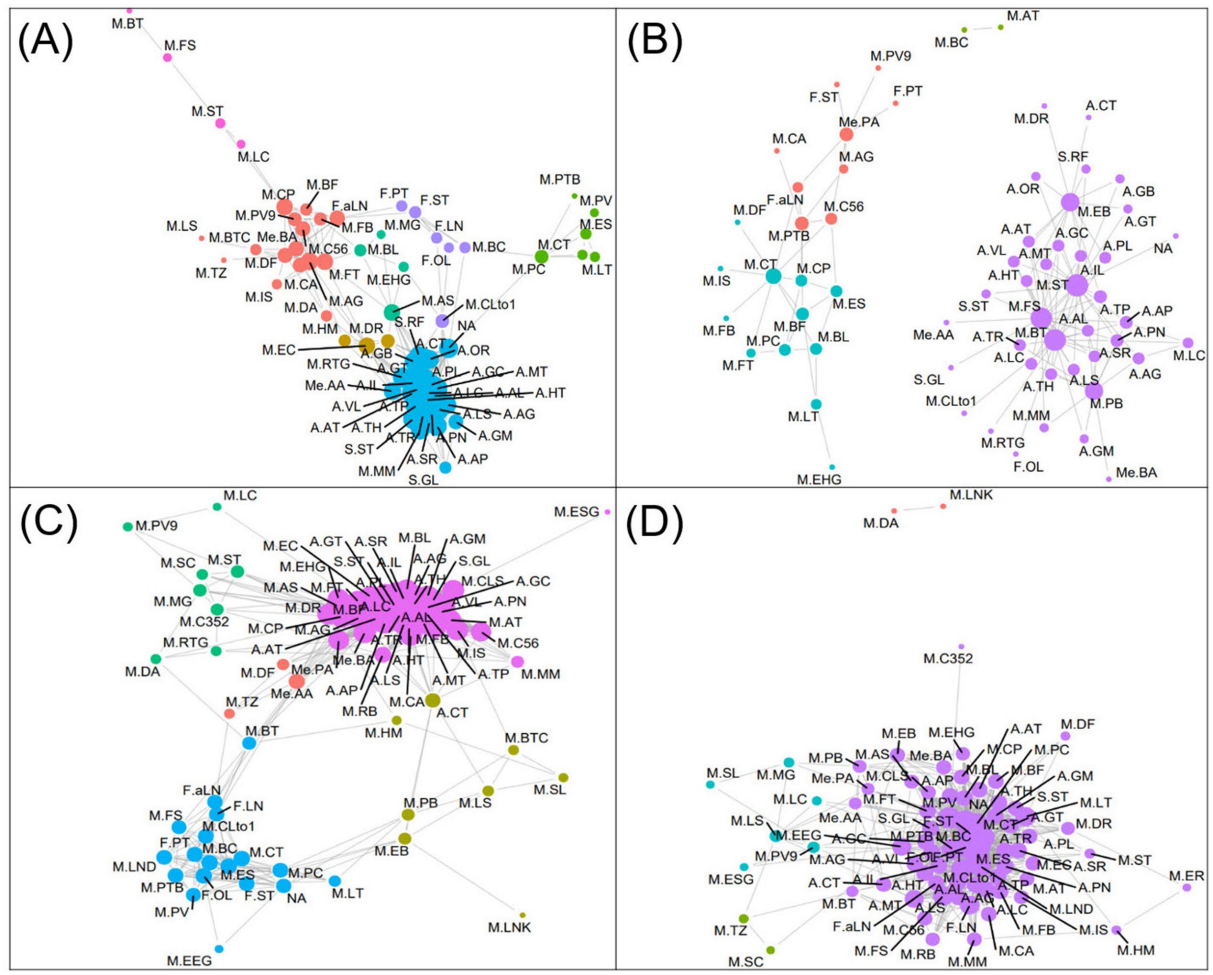
Positive **(A,C)** and negative **(B,D)** network analysis between the nutrient contents, microbial abundance changes (0 h–12 h), and metabolite contents produced by microbiota. The size of the nodes is indicative of the centrality of each variable, while the edges represent the correlation linkage. S.GL, glucose; S.ST, stachyose; S.RF, raffinose; A.GM, glutamine; A.LS, lysine; A.SR, serine; A.PN, phenylalanine; A.AP, asparagine; A.AG, arginine; A.TR, tyrosine; A.MT, methionine; A.AL, alanine; A.LC, leucine; A.TH, threonine; A.HT, histidine; A.TP, tryptophane; A.IL, isoleucine; A.AT, aspartate; A.OR, ornithine; A.CT, citrulline; A.GT, glutamate; A.GB, GABA; A.PL, proline; A.VL, valine; F.OL, oleic acid; F.LN, linoleic acid; F.ST, stearic acid; F.PT, palmitic acid; F.aLN, *α*-linolenic acid; M.AG, agathobacter; M.AT, alistipes; M.AS, anaerostipes; M.BC, bacillus; M.BT, bacteroides; M.BF, bifidobacterium; M.BL, blautia; M.BTC, butyricoccus; M.C352, CAG-352; M.C56, CAG-56; M.CA, catenibacterium; M.CT, citrobacter; M.CLto1, clostridium sensu stricto1; M.CLS, collinsella; M.CP, coprococcus; M.DF, desulfovibrio; M.DA, dialister; M.DR, dorea; M.EB, enterobacter; M.EC, enterococcus; M.ER, erysipelotrichaceae UCG-003; M.ES, escherichia-shigella; M.FB, faecalibacterium; M.FT, fusicatenibacter; M.FS, fusobacterium; M.HM, holdemanella; M.IS, incertae sedis; M.LC, lachnoclostridium; M.LS, lachnospira; M.LND, lachnospiraceae ND3007 group; M.LNK, lachnospiraceae NK4A136 group; M.LT, lactococcus; M.MM, megamonas; M.MG, monoglobus; NA, not identified; M.PB, parabacteroides; M.PTB, phascolarctobacterium; M.PV, prevotella; M.PV9, prevotella_9; M.PC, pseudocitrobacter; M.RB, romboutsia; M.SC, streptococcus; M.SL, subdoligranulum; M.ST, sutterella; M.TZ, tyzzerella; M.EEG, *eubacterium eligens* group; M.EHG, *eubacterium hallii* group; M.ESG, *eubacterium siraeum* group; M.RTG, ruminococcus torque group; Me.AA, acetic acid; Me.PA, propionic acid; Me.BA, butyric acid.

A network analysis was conducted to explore positive interrelationships between nutrient content of eight digested pulses, microbiome changes, and metabolite production in the pulses after 12-h colonic fermentation (Figure 6A). The analysis revealed significant interconnectivity among nutrients, particularly amino acids and sugars, which exhibited high centrality and strong positive correlations.

In terms of changes in the microbiome, two major communities were identified. The first community, comprising *Faecalibacterium*, *Bifidobacterium*, *Agathobacter*, *Fusicatenibacter*, *Butyricoccus*, *Desulfovibrio*, *Catenibacterium*, *Coprococcus*, and *Prevotella 9*, demonstrated complex positive correlations both within the group and with nutrient components. Notably, *Fusicatenibacter*, *CAG-56*, and *Bifidobacterium* showed positive correlations with *α*-linolenic acid (ALA), while *Anaerostipes* was influenced by amino acids, subsequently affecting the growth of *Blautia*, *Monoglobus*, and *Fusicatenibacter*. The second community, centered around *Pseudocitrobacter*, including *Lactococcus*, *Prevotella*, *Phascolarctobacterium*, *Escherichia-Shigella*, and *Citrobacter*, no strong correlations with the nutrients were observed. Among the metabolites, acetic acid exhibited strong positive correlations with amino acids and *Enterococcus*, while butyric acid was more strongly associated with microbial changes than nutrient components.

Further analysis of RB, MB, and HS samples, which showed markedly increased microbial diversity, revealed distinct patterns (Figure 6C). In comparison to the complete network, the *Pseudocitrobacter*-centered and fatty acid-based communities merged in these samples, displaying strong direct correlations between fatty acids and microorganisms. Nutrient-microbe interactions were more frequent, indicating dense positive effects of nutrients on multiple microorganisms. Additionally, three metabolites had strong positive relations with amino acids and were particularly influenced by fatty acids such as α-linolenic acid.

In conclusion, the network analysis for RB, MB, and HS illustrated higher connectivity between communities and more extensive microbe-nutrient interactions compared to the complete network. This increase connectivity and interaction may explain the elevated microbial diversity observed in these three pulses, highlighting the complex interplay between nutrient composition and microbial ecology in pulse digestion and fermentation processes.

#### 3.3.2 Negative correlation networks

The negative network revealed two distinct communities, with the most significant centrality observed in a community dominated by *Bacteroides*, *Fusobacterium*, *Sutterella*, and *Enterobacter*. These four bacteria were commonly associated with eight amino acids: aspartate, glycine, histidine, isoleucine, methionine, proline, tryptophan, and valine. *Sutterella* and *Enterobacter* also exhibited negative correlations with glutamate, GABA, and ornithine. It is noteworthy that despite the known ability of these microorganisms to metabolize amino acids as a primary energy source, they demonstrated strong negative correlations with these nutrients.

On the other hand, fewer microorganisms showed negative correlations with fatty acids. Notable examples included *Citrobacter* and *Phascolarctobacterium*, which negatively correlated with *α*-linolenic acid, and *Bacteroides*, which showed a negative association with oleic acid. Among metabolites, propionic acid had negative correlations with *Agathobacter*, *CAG-56*, and *Prevotella_9*, as well as with fatty acids such as α-linolenic acid, palmitic acid, and stearic acid. Additionally, acetic acid and butyric acid had direct negative correlations with *Fusobacterium* and *Parabacteroides*.

The negative correlation network analysis for RB, MB, and HS samples (Figure 6D) revealed a different pattern compared to the overall network. In these samples, there was no clear community separation, and the centrality of fatty acids was the most prominent. Unlike the general network (Figure 6B), strong negative correlations were observed between fatty acids and amino acids in RB, MB, and HS. This analysis also supported the findings from the positive network, indicating that the nutrient composition of RB, MB, and HS digesta played a significant role in negatively influencing the growth of several microorganisms. These results emphasize the complex and counterintuitive relationships between nutrients and microbial communities in pulse digestion and fermentation processes.

#### 3.3.3 The linear relationship between nutrient composition and microbial diversity

In fact, nutrient analysis of the digesta from RB, MB, and HS demonstrated relatively small differences in fatty acid and amino acid content compared to the other pulses. For example, the average range of fatty acid content in the digesta of RB, MB, and HS was 0.04–0.16, 0.64–5.07, 0.22–1.70 μg, respectively, while KB, CB, and BB showed similar fatty acid content (0.82–5.51, 0.55–4.22, 0.41–3.10 μg) (Supplementary Table S3). However, the average amino acid content of KB, CB, and BB was significantly higher (1.85–20.54, 1.65–14.99, 0.10–92.05 μg) than that of RB, MB, and HS (0.24–10.38, 0.16–4.30, 0.14–2.78 μg) (Supplementary Table S2). Thus, it is suspected that a balanced nutrient profile, rather than one dominated by a single nutrient, may promote diverse and active microbial interactions.

**Figure 7:**
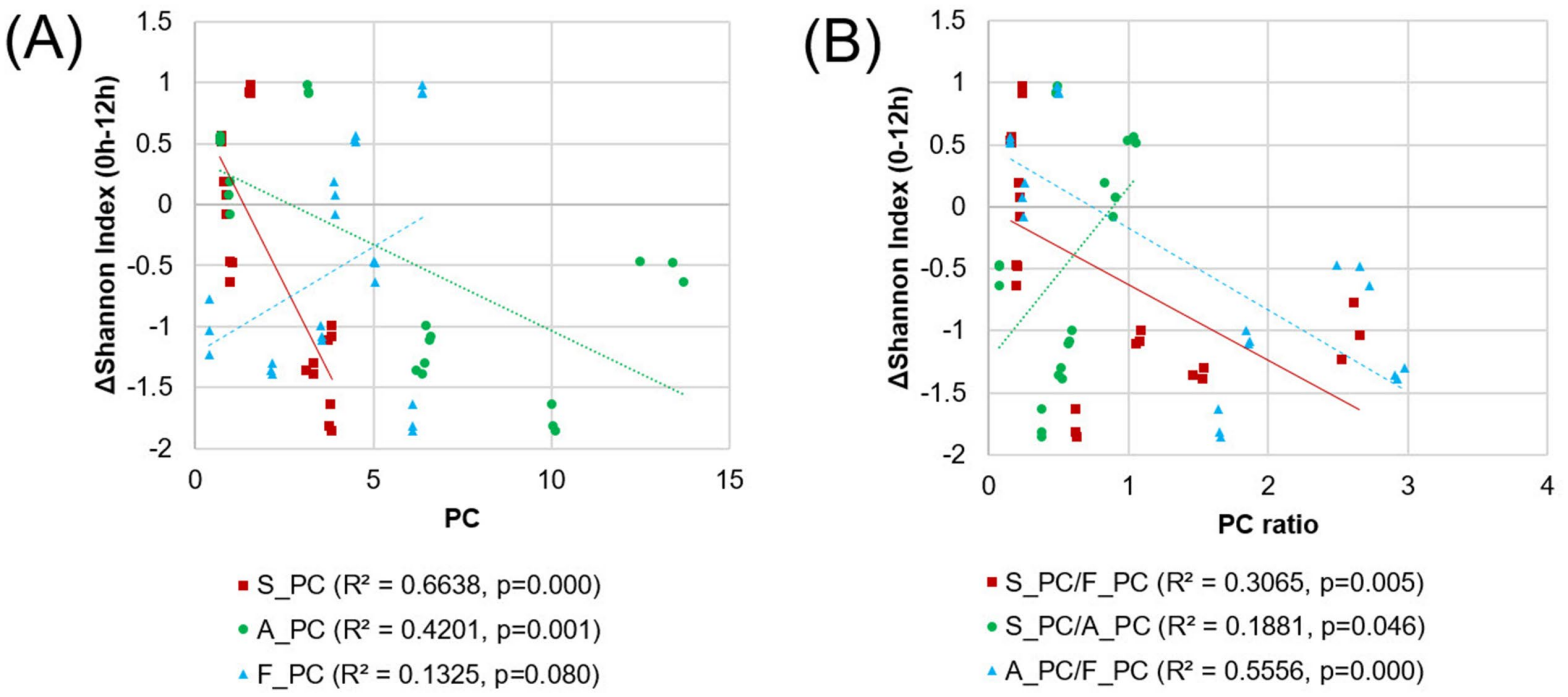
The linear regression analysis between the changes in the Shannon index for each pulse sample and the principal components of each nutrient **(A)** or their ratios **(B)**. The amino acid content data for SBs were identified as outliers and were subsequently excluded from the analysis.

To further investigate the quantitative relationship between nutrient balance and microbial diversity implied by the network analysis, linear regression analyses were performed (Figure 7). The coefficient of determination between changes in the SI from 0 h to 12 h and the principle component score (PC1) of each nutrient group (sugars, S_PC; amino acids, A_PC; fatty acids, F_PC) was examined. Results showed a strong inverse correlation between SI changes and sugars (R^2^ = 0.6638, *p* = 0.000) and a moderate correlation with amino acids (R^2^ = 0.4201, *p* = 0.001).

While the fatty acid content itself has a limited correlation with diversity (R^2^ = 0.1325, *p* = 0.080), its ratio with other nutrients significantly influenced the gut environment (Figure 7B). The ratio of S_PC to F_PC had a moderate negative correlation with the SI change (R^2^ = 0.3065, *p* = 0.005), while the A_PC to F_PC ratio was inversely related to SI change (R^2^ = 0.5556, *p* = 0.000). The S_PC to A_PC ratio had a weak positive correlation (R^2^ = 0.1881, *p* = 0.046). These results from quantitative analyses reinforce the findings from network analysis, implying the importance of nutrient balance in shaping microbial diversity.

## 4 Discussions

### 4.1 Health-affecting bacterial growth of *in vitro* gut fermentation samples

16S (V3-V4) rRNA sequencing and taxonomic profiling unveiled an enriched microbial community in RB, MB, and HS samples along with the fermentation time. Furthermore, there was a decrease in the relative abundance of health-negative genera such as *Escherichia-Shigella*, with an increase in beneficial bacteria including *Bacteroides* and *Eubacterium* groups. The term *Escherichia-Shigella* refers to both *Escherichia coli* and *Shigella*, which are genetically similar and difficult to be distinguished by 16S rRNA analysis. While most strains of *E. coli* are nonpathogenic and symbiotic in the human gut, *Shigella* is known to cause dysentery through intestinal infection (Wright et al., 2015; Xu et al., 2022). On the other hand, *Bacteroides*, although functionally diverse at the species level, plays a critical role in degrading complex carbohydrates in the gut, which supports intestinal immune function and homeostasis. Research has demonstrated that individuals with inflammatory bowel diseases (IBD), such as Crohn’s disease and ulcerative colitis, tend to have higher *Escherichia-Shigella* and lower *Bacteroides* levels (Wright et al., 2015).

*Eubacterium oxidoreducens* is known to participate in the oxidation–reduction reactions of various organic compounds in the gut and has been studied for its health-promoting role in cancer research, including esophageal (Li et al., 2024) and small cell lung cancer (Yang et al., 2024). *Eubacterium hallii* is an important gut bacterium that produces short-chain fatty acids (SCFAs), such as butyrate and propionate, which serve as an energy source for intestinal cells (Engels et al., 2016) and have been reported to contribute to anti-inflammatory effects and improved insulin sensitivity (Mukherjee et al., 2020; Seegers et al., 2021). *Eubacterium eligens*, known for its anti-inflammatory properties, regulates intestinal immune responses in the gut and may have protective effects against inflammatory bowel disease (IBD) (Chung et al., 2017; Olendzki et al., 2022). All of these species are known to metabolize complex carbohydrates and dietary fiber as energy sources, suggesting that measuring the dietary fiber and oligosaccharide content of RB may help identify the specific factors influencing the growth of these microorganisms.

While RB, HS, and MB positively influenced the distribution of a variety of microorganisms during colonic digestion, diversity analyses revealed that both *α*- and *β*-diversity varied among the initial fermentation samples (0 h). It has been previously reported that environments with high microbial diversity are known to be resilient to microbial perturbations, and ecological theory posits that environments with low microbial diversity are inherently less receptive to the colonization of new microbial species, as reduced diversity limits the availability of ecological niches and competitive opportunities (Lozupone et al., 2012; Bourdeau-Julien et al., 2023). Nevertheless, the present results, where samples with initially high diversity experienced a decrease while those with low diversity saw an increase, illustrated that RB, MB, and HS may have significant factors that promote a favorable gut environment compared to the others.

Not only was diversity enhanced in the three pulses, but there was also a recognized growth of multiple health-promoting microbiota including *Bacteroides, Bifidobacterium*, and *Coprococcus*. *Bacteroides* spp. protect the gut microbiome from pathogens and provide an energy source (Zafar and Saier, 2021). Approximately 50% of the carbohydrate in pulses is starch, which is converted to resistant starch (RS) during heat treatment. The metabolic capabilities of the gut microbiota vary depending on structural differences in RS, with RS4 consumption specifically known to increase the abundance of *Bacteroides* and *Bifidobacterium* (Kadyan et al., 2022).

Meanwhile, SCFA-producing *Coprococcus* (Nogal et al., 2021) has been found to grow with high quality diets. Since SCFAs are produced by the metabolism of anti-inflammatory bacteria that participate in energy synthesis in the human body, increasing the abundance of *Coprococcus* may help the growth of beneficial gut microbiomes (Laitinen and Mokkala, 2019). *Coprococcus* and *Lachnoclostridium*, along with *Lachnospira*, belong to the family *Lachnospiraceae*, which, according to NCBI, consists of 58 genera and several unclassified strains (Vacca et al., 2020). *C. comes*, consisting of 86% of *Coprococcus* genera, is a beneficial butyrate-producing bacterium found in the human gut. Butyrate, one of its major metabolic byproducts, plays a pivotal role in maintaining the intestinal barrier, reducing inflammation, and facilitating gut health. Studies have linked *C. comes* to positive outcomes in mental health, particularly in reducing symptoms of depression, due to its involvement in the gut-brain axis (Notting et al., 2023). Moreover, butyrate production by *C. comes* has been associated with anticancer properties, particularly in protecting against colorectal cancer by supporting the gut epithelium and regulating inflammatory processes (Liu et al., 2024). *Clostridium symbiosum*, the only species detected in the genus *Lachnoclostridium*, contributes to the fermentation of dietary fiber and aids in the production of SCFAs such as butyrate, which supports gut barrier function and reduces inflammation. By producing SCFAs, it can regulate metabolism and immune responses, playing a protective role in metabolic conditions like type 2 diabetes and colorectal cancer (Wang et al., 2012; Ren et al., 2024). However, overgrowth of *C. symbiosum* has been observed in conditions of gut dysbiosis, which may be associated with diseases such as obesity and inflammatory bowel disease (IBD) (Rodríguez et al., 2020).

The fermentation of MB digesta led to an enhanced relative abundance of *Odoribacter*, while HS digesta promoted the proliferation of *Butrycimonas*. Previous studies have indicated that *Odoribacter*, *Butyricimonas*, and *Bacteroides* are positively related with human cognitive function and contribute to the anti-inflammatory pathway and the modulation of the immune system (Zeng et al., 2021). The beneficial gut bacterium *O. splanchnicus* is known to utilize dietary fiber and resistant starch as its primary energy source, producing short-chain fatty acids (SCFAs) (Hiippala et al., 2020; Ren et al., 2024). During the fermentation of RB and MB, *Alistipes* showed a significant increase, with *Alistipes onderdonkii* emerging as the predominant species within this genus, and is recognized as a characteristic member of the gut microbiome with anti-inflammatory properties and involvement in the inhibition of tumor necrosis factor (TNF). It has been reported to play a beneficial role in cancer immunotherapy by modulating the tumor microenvironment (Parker et al., 2020; Li et al., 2023).

The genera *Sutterella* and *Enterobacter*, which showed increased abundance in HS and MB, and HS and RB respectively, are gut microbes with similar potential impacts on human health. *Sutterella* has been associated with inflammatory conditions such as irritable bowel syndrome (IBS) and autism spectrum disorders, suggesting that it may play a role in gut inflammation and immune modulation (Hiippala et al., 2016). On the other hand, *Enterobacter* species are opportunistic pathogens causing infection and dysbiosis, especially in immunocompromised individuals (Moreira de Gouveia et al., 2024). Both genera can affect gut homeostasis and contribute to disease states.

Besides, the relative abundance of *Akkermansia* significantly increased in HS and MB. *Akkermansia muciniphila* is one of the most abundant single species in the human gut microbiota, comprising 0.5–5% of the total bacteria. It degrades the mucin layer of the intestinal mucosa and produces beneficial metabolites such as acetate and propionate. Through this mechanism, *A. muciniphila* has been shown to reduce body fat mass and adipose tissue inflammation while improving insulin sensitivity, thereby enhancing gut barrier function (Cani and de Vos, 2017).

### 4.2 Nutrient-microbiome-metabolite network claims importance of nutrient balance for gut health

Protein degradation during pulse digestion led to a consistent increase in amino acid levels. The high interconnectivity between amino acids and sugars observed in the network analysis suggested that the pulses used in the experiment had proportional amounts of protein and carbohydrates. Fatty acids did not have direct positive networks with amino acids or sugars, indicating that the fat content of the pulses does not necessarily align with protein or carbohydrate level and may vary among the pulses.

In the positive correlation network of the eight pulses, few links were observed between microbes and nutrients. *Fusicatenibacter*, *CAG-56*, and *Bifidobacterium* were positively correlated with the fatty acid *α*-linolenic acid (ALA), while *Anaerostipes* was influenced by amino acids and associated with the growth of *Blautia*, *Monoglobus*, and *Fusicatenibacter*. Coakley et al. (2009) reported the ability of *Bifidobacterium* strains to convert linoleic acid to c9, t11-conjugated linoleic acid (CLA) and grow in the presence of anti-inflammatory ALA. Since the anti-inflammatory properties may create a favorable gut environment that is likely to foster the proliferation of SCFA-producing bacteria (Yang et al., 2023), the growth of SCFA-producing *Fusicatenibacter* (Pihelgas et al., 2024) might have been affected by ALA. *Anaerostipes*, a butyrate-producing genus, interacts with amino acids such as glutamate and lysine, which serve as substrates for SCFA production, especially butyrate, known for its anti-inflammatory effects (Singh et al., 2023) which can in turn promote the growth of *Fusicatenibacter*.

Cross-feeding interactions explain the correlations within this bacterial community, where one microbe’s byproducts support the growth of others. These interactions, several bacteria degrade dietary components into intermediate metabolites such as acetate or lactate, which are then sequentially used as nutrient substrates by butyrate-producing bacteria. Rivière et al. (2016) reported relationships between acetate- or lactate-producing *Bifidobacterium* and butyrate-producing colonic bacteria, such as *Faecalibacterium prausnitzii* (clostridial cluster IV) and *Anaerostipes*. Accordingly, positive associations of the SCFA metabolite content with microbial alteration were more frequent than those with nutrient content. This may stress that the composition and activity of the gut microbiota are of paramount importance in determining the production of key metabolites.

Therefore, optimizing microbial conditions may yield greater advantages to gut health than focusing solely on individual nutrient contents. Recent studies have highlighted the potential of dietary interventions in modulating the gut microbiome by enhancing beneficial microbial populations or increasing the production of SCFA. For instance, Purdel et al. (2023) reported that fasting and/or time-restricted regimens can achieve gut health. Time-restricted fasting, intermittent fasting, and Ramadan fasting protocols were shown to increase *Akkermansia muciniphila* and *Bacteroidetes*, associated with improved metabolic health and reduced gut inflammation. Caloric restriction and alternate day fasting resulted in increased microbial diversity and promoted *Bacteroides* and SCFA production. In addition, water-only fasting and fasting-mimicking diets led to a high abundance of beneficial microbes like *Lactobacillus* and *Bifidobacterium*, improving immune function and gut health.

The synergistic role of prebiotics―indigestible dietary fibers that provide food for beneficial bacteria―with probiotics in promoting a diverse and stable gut microbiome has also been emphasized (Shang et al., 2024). Combining probiotic-rich meals with prebiotic fibers enhances intestinal permeability defenses through SCFA production, maintaining stability in gut flora despite dietary or environmental changes. A previous study by Starke et al. (2023) reported that amino acid-cross-feeding bacteria contribute to the compositional stability of the gut microbiome, with no evidence that dietary amino acid intake affects the frequency of auxotrophy, suggesting that simple manipulation of nutrient content may not be sufficient to regulate microbial-nutrient metabolic pathways.

In the negative network analysis of the eight pulses, a community centered around *Bacteroides*, *Fusobacterium*, *Sutterella*, and *Enterobacter* exhibited the greatest centrality and had strong correlations with amino acids. These findings were on the opposite side with previous studies that have identified various taxa involved in amino acid metabolism, including nitrogen metabolism and the tricarboxylic acid (TCA) cycle (Kanehisa et al., 2023), implying the positive relationships between those microbes and amino acids. For example, some *Enterobacter* and *Sutterella* species are engaged in the glutamate metabolism of the TCA cycle, producing glutamine as a byproduct. The pathway also includes the conversion of glutamate to GABA, suggesting that a decrease in glutamate might lead to a reduced GABA level (Kanehisa et al., 2023). Given that no relationships were recognized for these bacteria with amino acids even in the positive network of present study, it was discerned that an imbalance in the overall nutritional state provoked by amino acids may have created an unfavorable gut environment for bacterial growth. Despite their reliance on amino acids for energy, their metabolism can produce toxic byproducts such as ammonia and hydrogen sulfide. These substances can disrupt gut homeostasis, contribute to dysbiosis, and negatively affect the balance of microbial populations (Lin et al., 2017).

### 4.3 Quantitative relationships among carbohydrates, amino acids, and fatty acids modulating the gut environment depend on context-rule

The positive and negative networks for RB, MB, and HS had higher connectivity between communities, with a broader range of microbes interacting directly or indirectly with fatty acids and amino acids compared to the complete network. This could interpret the improved microbial diversity observed in these three pulses. Since the three pulses comprising the subset network were not identified for their nutrient abundance, it was proposed that the overall nutritional status in RB, MB, and HS influenced the nutrient-microbe interactions.

Evidence from this study insists that facilitating a gut environment driven by the comprehensive nutrient balance, rather than individual nutrients, promotes eubiosis, thereby enhancing microbial diversity and stimulating the growth of beneficial microorganisms. In fact, the linear regression demonstrated the quantitative relationship between nutrient content and microbial diversity. Excess sugars and amino acids were found to negatively affect the gut environment, with a weak correlation between fatty acids and the SI. However, fatty acid content as a ratio to sugars or amino acids significantly influenced gut microbial diversity. Consequently, the nutritional context-dependence for maintaining gut health was highlighted by elucidating the importance of avoiding overconsumption of sugars and amino acids while maintaining a balance with fatty acids. In the human colon, an overload of certain nutrients can promote the overgrowth of specific microorganisms, which can disrupt the environment for others and reduce overall microbial diversity. Amino acids, in particular, can facilitate the growth of pathogenic amino-acid-degrading bacteria, such as *Clostridium*, and produce metabolites such as ammonia, phenols, and amines that can alter gut pH.

A previous opinion by Rios-Covian et al. (2017) suggested the metabolic remodeling of the host and *Bacteroides* in response to dietary organic nitrogen/carbohydrate combinations. In addition, a study in mice by Holmes et al. (2017) described that the pattern of protein and carbohydrate intake drives diet-microbiome interactions. The interdependence of sugars and amino acids in the digested pulses used in this study obscured the association of the ratio of carbohydrates to amino acids with microbiota, which also emphasized the relevance of the context of the food matrices in determining microbiome metabolism. Although this study concluded that balancing fatty acids with sugars or amino acids is relevant to a healthy microbiome, the context-based effect may not be distinguished when examining food groups in which carbohydrates are predominant, such as fruits and vegetables. Therefore, elucidating the multidirectional connections between nutrients and microbes using a variety of food ingredients might notify the way to combine foods that complement each ingredient’s nutritional benefits for gut microbiota health.

Several limitations need to be discussed in further studies. Although the primary energy source of microorganisms – saccharides, amino acids, and fatty acids—was quantified in this study, the content of complex carbohydrates such as dietary fiber, resistant starch, or a variety of oligosaccharides also needs to be analyzed. Functional food components including isoflavones could also be the other candidates, as their effects on human microbiome changes were reported (Aboushanab et al., 2022; Narduzzi et al., 2022). Furthermore, since a single food group, pulses, was studied in the present research, other food groups with diverse matrices containing various proportions of macronutrients should be examined to demonstrate the multifaceted interactions between nutrient components and microbial diversity.

## 5 Conclusion

This study elucidated the effects of nutrient intake from pulse consumption on the human microbiome and metabolite changes. Following the application of *in vitro* digestion and fermentation to pulses, 16S rRNA analysis revealed a reduction in harmful pathogenic microorganisms and a specific increase in beneficial bacteria in the RB, HS, and MB samples. In addition, SI and Bray-Curtis distance calculations indicated that these pulses enhanced gut microbial diversity that differed from the others. Network analysis and linear regressions examining the interaction between changes in microbial communities and the nutrient content of each sample suggested that this effect was due to the balanced nutrient profiles of the three pulses. However, this should not be interpreted to mean that other pulses are detrimental to gut health. As the present study was primarily limited to pulse digesta, this can be overcome in real-life dietary contexts by consuming a variety of food groups beyond pulses. Nevertheless, our research demonstrated that maintaining a diet rich in nutrient-balanced foods, including RB, HS, and MB, supports gut microbial diversity and an environment conducive to the growth of health-positive bacteria, such as *Alistipes*, *Akkermansia*, *Coprococcus*, and *Eubacterium* groups.

This pioneering study quantitatively investigated the extensive interdependence between key nutrients and gut microbiota dynamics. By applying food ingredients in their consumed forms to the replicated model of human digestion and gut microbiota, their contribution to microbial shifts and metabolite production could be explored, reflecting real-world conditions. There is a need to further examine the effects of commonly consumed individual ingredients in everyday meals—such as meat, fish, dairy products, fruits, and vegetables—on the gut microbiota. As more data accumulate, it may be possible to identify optimal food combinations for improving gut health. Additionally, *in vitro* fermentation model that simulates gastrointestinal conditions in patients with gut disorders could potentially provide tailored dietary recommendations for therapeutic purposes.

## Data Availability

The original contributions presented in the study are publicly available. This data can be found here: https://www.ncbi.nlm.nih.gov/, PRJNA1212984.
