# Supplementary material for: Macronutrient balance determines the human gut microbiome eubiosis: insights from in vitro gastrointestinal digestion and fermentation of eight pulse species
Source: Front Microbiol. 2025 Jan 30;15:1512217. doi: 10.3389/fmicb.2024.1512217 (PMC11823474; doi:10.3389/fmicb.2024.1512217)
Supplement: Supplementary file 1 [file Data_Sheet_1.PDF]

Table S1. Free saccharides content (μg) in the digested samples of eight pulse species

Sample / Content (μg)	Soybean	Kidney bean	Cowpea	Chickpea	Blackbean	Mungbean	Red bean	Heunguseul
Stachyose	16.15±0.99^{c}	36.03±3.80	63.84±1.80^{a}	58.56±0.99^{a}	42.17±0.45	9.05±0.54^{b}	12.29±0.36^{bc}	8.17±0.64^{b}
Raffinose	-	-	-	-	9.14±0.57	-	-	-
Sucrose	-	-	-	-	-	-	-	-
Lactose	-	-	-	-	-	-	-	-
Glucose	18.88±0.22	246.77±1.93	206.96±0.77	228.95±1.24	-	22.66±0.79	105.96±0.53	8.10±0.27
Fructose	-	-	-	-	-	-	-	-

The values assigned to the same letter have no significant difference between pulses (ANOVA, Tukey's method p>0.05).

-: not detected (ND).

Table S2. Free amino acids content (μg) in the digested samples of eight pulse species

Sample / Content (μg)	Soybean	Kidney bean	Cowpea	Chickpea	Blackbean	Mungbean	Red bean	Heunguseul
Aspartate	8.50±0.14^{b}	7.63±0.07^{b}	10.51±0.11	12.65±0.11	23.64±0.95	1.76±0.03^{a}	4.80±0.05	0.80±0.01^{a}
Glutamate	23.53±0.36^{a}	20.54±0.19^{a}	23.80±0.26^{a}	30.76±0.13	92.05±3.93	4.30±0.09^{b}	10.38±0.11	2.78±0.04^{b}
Asparagine	11.93±0.29	9.81±0.11	8.97±0.10	18.54±0.07	8.36±0.34	0.97±0.02	3.03±0.01	0.34±0.00
Serine	11.35±0.21^{a}	11.38±0.12^{a}	10.96±0.11^{a}	17.09±0.07	12.92±0.57	1.01±0.02^{b}	3.56±0.02	0.47±0.00^{b}
Glutamine	16.13±0.25	10.50±0.14^{a}	10.33±0.13^{a}	18.42±0.08	0.23±0.01^{b}	0.79±0.03	4.22±0.04	0.14±0.00^{b}
Histidine	4.80±0.09^{a}	4.66±0.19^{a}	4.74±0.02^{a}	6.62±0.15	9.11±0.33	0.40±0.01^{b}	2.40±0.02	0.29±0.02^{b}
Glycine	5.31±0.15^{a}	5.89±0.08^{ab}	6.30±0.07^{b}	9.22±0.06	15.10±0.70	0.78±0.01^{c}	2.58±0.01	0.57±0.01^{c}
Threonine	6.58±0.12^{a}	7.10±0.08^{a}	8.24±0.11	9.46±0.09	10.44±0.39	0.95±0.02^{b}	3.01±0.03	0.45±0.01^{b}
Citrulline	-	-	-	-	7.43±0.33	-	0.24±0.00^{a}	0.22±0.01^{a}
Arginine	19.99±0.23	10.81±0.16^{a}	11.31±0.20^{a}	27.60±0.18	10.78±0.39^{a}	1.20±0.03	6.02±0.04	0.48±0.01
Alanine	8.00±0.15	7.24±0.10^{a}	7.23±0.07^{a}	12.62±0.08	13.83±0.58	0.80±0.01^{b}	2.93±0.02	0.64±0.00^{b}
GABA	-	-	-	-	0.10±0.00	-	-	-
Tyrosine	8.66±0.09	7.61±0.18^{a}	7.90±0.13^{a}	11.02±0.05	11.80±0.49	0.77±0.02^{b}	3.86±0.04	0.41±0.01^{b}
Valine	9.21±0.15	10.38±0.22^{a}	10.56±0.08^{a}	12.45±0.02	20.39±0.84	1.27±0.02^{b}	5.09±0.05	0.75±0.00^{b}
Methionine	2.53±0.06	1.85±0.06^{a}	1.65±0.04^{ab}	4.33±0.01	5.55±0.24	0.16±0.00^{c}	1.36±0.01^{b}	0.19±0.00^{c}
Tryptophan	3.71±0.07	3.00±0.08^{a}	3.16±0.04^{a}	4.40±0.03	6.40±0.21	0.31±0.02^{b}	1.34±0.02	0.17±0.01^{b}
Phenylalanine	11.42±0.20^{a}	10.47±0.12^{a}	10.63±0.10^{a}	21.76±0.19	17.57±0.73	0.99±0.02^{b}	4.92±0.04	0.56±0.01^{b}
Isoleucine	7.89±0.10	9.27±0.09^{a}	9.65±0.10^{a}	10.97±0.13	21.92±0.92	1.10±0.02^{b}	4.56±0.04	0.56±0.01^{b}
Ornithine	-	-	-	-	1.93±0.12	-	-	-
Leucine	15.78±0.22^{a}	15.21±0.22^{a}	14.99±0.13^{a}	23.39±0.09	27.62±1.19	1.49±0.03^{b}	7.20±0.06	0.72±0.00^{b}
Lysine	11.75±0.32	9.45±0.31^{a}	9.92±0.20^{a}	16.22±0.34	10.12±0.45	0.95±0.06^{b}	5.71±0.04	0.98±0.03^{b}
Proline	7.47±0.07^{a}	6.69±0.26^{a}	6.32±0.26^{a}	11.00±0.55	0.45±23.44	1.08±0.02^{b}	3.01±0.04	0.62±0.01^{b}

The values assigned to the same letter have no significant difference between pulses (ANOVA, Tukey's method $p>0.05$).

-: not detected (ND).

Table S3. Free fatty acids content(µg) in the digested samples of eight pulse species

Sample / Content(µg)	Soybean	Kidney bean	Cowpea	Chickpea	Blackbean	Mungbean	Red bean	Heunguseul
Palmitic acid	2.94±0.00	3.67±0.00	2.40±0.00	0.48±0.00	1.02±0.00	2.62±0.01	0.16±0.00	1.70±0.00
Stearic acid	1.00±0.00	0.82±0.01[a]	0.55±0.01	0.14±0.00	0.41±0.01	0.64±0.01	0.10±0.00	0.81±0.01[a]
Oleic acid	10.02±0.01	2.59±0.00	1.59±0.00	0.50±0.00	2.04±0.02	0.70±0.01	0.04±0.00	0.74±0.01
Linoleic acid	25.27±0.01	5.39±0.01	4.15±0.01	0.56±0.00	3.10±0.02	5.07±0.03	-	1.15±0.00
α-linolenic acid	3.36±0.00	5.51±0.01	4.22±0.01	-	0.45±0.00	1.53±0.01	-	0.22±0.00

The values assigned to the same letter have no significant difference between pulses (ANOVA, Tukey's method $p>0.05$).

-: not detected (ND).
